# Case study on the pathophysiology of Fabry disease: abnormalities of cellular membranes can be reversed by substrate reduction *in vitro*

**DOI:** 10.1042/BSR20160402

**Published:** 2017-04-28

**Authors:** Graham Brogden, Hadeel Shammas, Katia Maalouf, Samara L. Naim, Gabi Wetzel, Mahdi Amiri, Maren von Köckritz-Blickwede, Anibh M. Das, Hassan Y. Naim

**Affiliations:** 1 Department of Physiological Chemistry, University of Veterinary Medicine Hannover, Buenteweg 17, 30559 Hannover, Germany; 2 Fish Disease Research Unit, University of Veterinary Medicine Hannover, Buenteweg 17, D-30559 Hannover, Germany; 3 Department of Paediatrics, Hannover Medical School, Carl-Neuberg-Strasse 1, D-30625 Hannover, Germany; 4 Faculty of Science, University of Fribourg, Switzerland

**Keywords:** lipid rafts, lysosomal storage disease, lipids, membranes

## Abstract

It is still not entirely clear how α-galactosidase A (GAA) deficiency translates into clinical symptoms of Fabry disease (FD). The present communication investigates the effects of the mutation N215S in FD on the trafficking and processing of lysosomal GAA and their potential association with alterations in the membrane lipid composition. Abnormalities in lipid rafts (LRs) were observed in fibroblasts isolated from a male patient with FD bearing the mutation N215S. Interestingly, LR analysis revealed that the distribution of cholesterol and flotillin-2 are distinctly altered in the Fabry fibroblasts when compared with that of the wild-type cells. Furthermore, increased levels of glycolipid globotriaosylceramide 3 (Gb3) and sphingomyelin (SM) were observed in non-raft membrane fractions of Fabry cells. Substrate reduction with *N*-butyldeoxynojirimycin (NB-DNJ) *in vitro* was capable of reversing these abnormalities in this patient. These data led to the hypothesis that alterations of LRs may contribute to the pathophysiology of Morbus Fabry. Furthermore, it may be suggested that substrate reduction therapy with NB-DNJ might be a promising approach for the treatment of GAA deficiency at least for the selected patients.

## Introduction

Fabry disease (FD, MIM ID # 301500) also known as Anderson disease in the Anglo-American literature is a multisystemic disease caused by deficiency of the lysosomal enzyme α-galactosidase A (GAA) [1]. It is inherited as an X-linked trait, thus, the phenotype is generally more severe in male hemizygotes as compared with female heterozygotes [2]. Characteristic clinical features are pain crises, acroparesthesias, angiokeratoma, cornea verticillata, cardiac dysfunction, kidney disease, cerebrovascular events, hearing difficulties and gastrointestinal symptoms [3].

As a result of GAA deficiency, biodegradation of glycosphingolipids is compromised, which leads to the intralysosomal accumulation of terminal galactosyl containing neutral lipids [4]. Lipid storage is not limited to lysosomes, elevated concentrations of glycosphingolipids were even found in plasma [5]. Storage phenomena were observed in the placenta of affected pregnancies [6], however clinical symptoms set in with a delay of several years in most patients [2,3]. The concept that clinical symptoms are directly related to storage of glycolipids resulting in ischaemia of organs is at best simplistic indicating that other functional disturbances must occur.

In a previous study, we have shown that trafficking of a representative protein associated with detergent-resistant membranes (DRM)/lipid rafts (LRs), namely dipeptidyl peptidase IV (DPPIV), to the cell surface was hampered in fibroblasts from a Fabry patient, which may lead to altered signalling and transport at the cell surface [7]. LRs are distinct, ordered, liquid domains enriched in sphingolipids and cholesterol segregated from less ordered membrane domains composed of mainly unsaturated phospholipids. The function of LRs depends on their lipid membrane composition. There is increasing evidence for a regulatory role of LRs particularly in signalling and trafficking pathways, however, further research is required to determine the effects of trafficking disorders on the pathology of FD [8–11]. It is still not clear if therapeutic interventions in FD can completely reverse secondary biochemical alterations, particularly changes in LRs organization.

In the present study, we characterize alterations of LRs in fibroblasts derived from a male Fabry patient bearing the mutation N215S in comparison with the wild-type cells. By using substrate-reduction therapy (SRT) with *N*-butyldeoxynojirimycin (NB-DNJ or miglustat) *in vitro*, we address the question whether reduced lysosomal storage of glycolipids leads to a reversal of biochemical abnormalities in cellular membranes of this patient. To the best of our knowledge, this is the first study demonstrating in a patient with FD the ability of substrate reduction therapy *in vitro* to restore LR alterations.

## Materials and methods

### Patient

A fibroblast cell culture from an adult male with FD (approximately at nucleotide 644, A>G N215S) from our outpatient clinic was used here. The cell line was established from a skin biopsy taken from the thigh primarily for diagnostic purposes, and the patient gave consent that the cell cultures can be used for scientific studies.

Clinically, the patient presented an attenuated course with fatigue, arrhythmia, cardiac hypertrophy, hypothyroidism, vertigo and he required a pacemaker. Age matched control cells were taken from our tissue bank. The study was approved by our local ethics review board at Hannover Medical School, Germany.

### Cell culturing

Skin fibroblasts were grown in culture as previously described [7]. Treatment with 50 mM NB-DNJ was performed for 72 h, renewed in fresh medium every 24 h and cultured in Dulbecco’s Modified Eagle’s Medium, low glucose with 10% FBS and added penicillin and streptomycin at 37°C in 5% CO_2_.

### Immunocytochemistry

Cells were seeded on coverslips coated with 1% gelatin, fixed with 4 % PFA for 10 min on ice followed by 20 min at 22°C and quenched with 50 mM ammonium chloride for 30 min at 22°C. The coverslips were then permeabilized with 0.2% Triton X-100 for 30 min at 22°C and blocked with a solution containing 1% BSA, with added 0.5% saponin, 0.1% Triton X-100 for additional permeabilization for 10 min at 22°C. Cells were then incubated with anti-α-galactosidase A (Sigma, Germany) for 1 h at 22°C. Horseradish peroxidase–conjugated goat anti-rabbit was used as a secondary antibody to visualize the GAA protein. Evaluation of slides was done using a Leica DMI6000CS confocal microscope with a HCXPLAPO 40× 0.75–1.25 oil objective. The gain setting remained constant throughout.

### Protein solubilization, LRs isolation and Western blot analysis

Total cell lysates were prepared by solubilizing the cells in 25 mM Tris buffer (pH 8) containing 0.5% sodium deoxycholate, 0.5% Triton X-100 and 50 mM NaCl for 30 min at 4°C followed by centrifugation at 10000 ***g*** for 10 min at 4°C to remove the debris. Protein concentration of the lysate was determined according to the Bradford method [12]. Wherever indicated, 50 μg of the lysate was treated with 0.5 μl (5 U/ml) of endoglycosidase H (endo H; Roche) for 1.5 h at 37°C.

For LRs isolation, cells were solubilized with 1% Triton X-100 and then subjected to sucrose density gradient fractionation as described before [13]. After ultracentrifugation, ten fractions were collected at 4°C from top to bottom. Sucrose was measured by a Hanna Instrument HI 96801 refractometer and expressed as sucrose concentration based on the Brix scale. The characterization of the sucrose density gradient showed that the increase in sucrose concentration between fraction 1 and 2 was approximately 12%, with fraction 1 and 2 containing 12.45 and 24.6% sucrose respectively (Supplementary Figure S1).

All the lysis steps were carried out in the presence of a mixture of protease inhibitors [14]. Equal volume from each fraction for LRs analysis or protein amounts from the total lysates were resolved by SDS/PAGE and transferred to PVDF membranes (Roche Diagnostics). Immunoblotting was performed as described before using primary antibodies against flotillin-2 (Santa Cruz Biotechnology, Germany), RhoA (Santa Cruz Biotechnology, Germany) and α-galactosidase A (Sigma, Germany) and then HRP–conjugated secondary antibodies [15]. Protein bands were visualized using ECL with SuperSignal West Femto Chemiluminescent Substrate (Thermo Scientific) and the ChemiDoc system (Bio–Rad, Germany).

### Cell surface biotinylation assay

Cell surface biotinylation was conducted according to Liu et al. [16]. Cells were then lysed with the lysis buffer mentioned above supplemented with protease inhibitors. DPPIV was immunoprecipitated from the lysate using a monoclonal antibody [17] and further processed by non-reducing SDS/PAGE followed by immunoblotting with streptavidin-HRP as described above.

### Lipid analysis

Fibroblasts isolated from a Fabry patient (containing the mutation N215S) and wild-type cells were cultured in 60 × 15 mm diameter Petri dishes. After 72 h of incubation with NB-DNJ, the cells were washed with PBS, scraped from the plate and resuspended in 1 ml PBS. The cells were then counted and adjusted to 300000 cells per sample. The samples were then pelleted at 800 × ***g*** for 10 min and resuspended in 1 ml 1% Triton X-100 in PBS. Lysis was completed by passing lysates through a 45-mm cannula syringe 15 times and overnight rotation at 4°C. Samples were then centrifuged for 30 min at 13000 × ***g*** at 4°C to remove cell debris. The supernatant was centrifuged at 100000 × ***g*** for 90 min at 4°C and finally the pellet and supernatant fractions were harvested and prepared for lipid analysis as previously described [18] or stored at –20°C for future analysis. Glycolipid and sphingomyelin (SM) analysis were performed based on previously published methods [18]. Briefly, for glycolipid analysis samples were resuspended in 250 μl chloroform/methanol solution and loaded dropwise on to a TLC silica gel 60 F_254_ plate (Merck). These plates were then run in a solution of chloroform, methanol and distilled water in a ratio of 65:35:8. The plates were developed in a solution containing 5% orcinol monohydrate (Sigma), 10% sulphuric acid and 85% distilled water. The plates were then baked for 10 min at 115°C. For the following lipids, high performance TLC (HPTLC) analysis was performed on silica gel 60 plates (Sigma): phosphatidylcholine (PC), phosphoethanolamine (PE), galactocerebroside (GC), SM, monoacylglycerol (MG), free fatty acids (FFA), cholesterol ester (CE), phosphatidylserine (PS), cardiolipin (CA). The samples were loaded in triplicates with 10 μl of each sample used. The HPTLC plates were then run in three separate running solutions. The first solution contained acetic acid, 1-propanol, chloroform, methanol and potassium chloride, the second contained n-hexane, diethyl ether and acetic acid, and the final running solution only contained 100% n-hexane. The plates were developed in a bath of copper sulphate solution containing 7.5% phosphoric acid and developed at 170°C. For statistical comparisons, samples were loaded in triplicates on the same plate to reduce interplate variation. Once cooled, the plates were scanned using a HP Scanjet G3010 scanner in 1594 × 785 pixel format and analysed using the CP ATLAS software (LazarSoftware) and the bands were quantified by intensity of pixels and identified by comparing them with a known standard based on the Rf value. Lipids were quantified within the linear range of the corresponding calibration curve and expressed as μg/3 × 10^5^ cells. Calibration curves for each lipid are included as a supplementary data file (Supplementary Figure S2). Quantities of the unknown glycolipid 1 (GL 1) were expressed as band intensity.

In a similar approach, lipids from sucrose density gradient fractions were isolated and their cholesterol content was analysed with a Hitachi Chromaster HPLC using a Chromolith® HighResolution RP-18 endcapped 100-4.6 mm column coupled to a 5-4.6 mm guard cartridge and heated to 32°C. Methanol was used as the mobile phase at a flow rate of 1 ml/min at 22 bar and a UV detector measuring at 202 nm to quantify the amount of cholesterol in each sample. The results are expressed as a percentage of the totals of fractions 1, 2, 3, 8, 9 and 10. Samples below the minimum detection limit were not included in the analysis.

### Quantifications and statistical analysis

Western blot bands were quantified using ImageJ software and lipid bands with CP Atlas (LazarSoftware). Data were analysed by Excel 2003 (Microsoft) and GraphPad Prism 6.0 (GraphPad Software). Lipid quantities between wild-type and FD fibroblasts were compared using Student’s *t* test for unpaired data. Comparisons between treated and untreated fibroblasts were performed using Student’s *t* test for paired data. A *P*-value ≤0.05 indicates a significant difference.

## Results and discussion

The present communication investigates the effects of the mutation N215S in FD on the trafficking and processing of lysosomal GAA and their potential association with alterations in the membrane lipid composition, particularly LRs and the subsequent trafficking of other membrane proteins. We further analysed the effects of NB-DNJ in restoring the membrane lipid composition suggesting a novel therapeutical approach for FD.

### The effect of the N215S mutation on the trafficking of GAA

**Figure 1:**
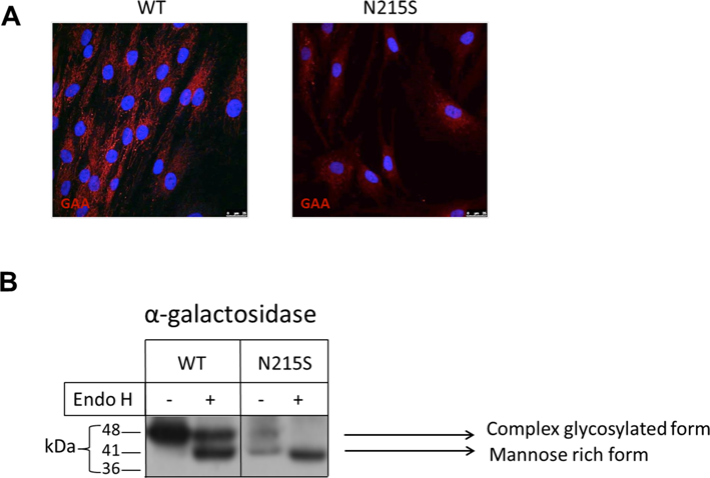
GAA mutations in the GAA-coding region affects its maturation and intracellular localization (**A**) GAA localization was visualized using immunocytochemistry. The nuclei are stained with DAPI prolong gold (blue) and GAA with anti-GAA and visualized with Alexa-Floura 568–conjugated secondary goat anti-rabbit (red). (**B**) Fibroblasts were solubilized in standard lysis buffer. Equal protein amounts were incubated with or without endo H, subjected to polyacrylamide SDS gel and followed by Western blotting, with GAA subsequently visualized using an anti-GAA antibody.

The consequences of the N215S mutation on maturation and trafficking of the GAA protein were investigated in fibroblasts isolated from a male Fabry patient and a healthy control. Immunochemistry revealed contrasting GAA locations: the net-like distribution depicts how the protein is distributed throughout the cell in the WT fibroblasts, whereas the more condensed distribution surrounding the nuclei suggests that the mutant protein is primarily associated with the endoplasmic reticulum in the fibroblasts containing the N215S mutation (Figure 1A).

Additionally, in support of the immunocytochemistry images, the endo H-digestion pattern clearly indicated that GAA in the wild-type fibroblasts has been trafficked from the ER and processed in the Golgi apparatus. Endo H was utilized to differentiate between the mature complex glycosylated GAA, appearing as a 48-kDa band and the immature mannose-rich, ER-located protein species, appearing as a 41-kDa band [19,20]. On the other hand, the mutation N215S in GAA resulted in the elimination of one potential N-glycosylation in the GAA protein, which harbours three N-glycosylation sites at positions 139, 192 and 215 of the primary sequence. Interestingly, two bands of variable glycosylation and intensity were detected in the Fabry fibroblasts, which were converted to one single band upon endo H treatment. This result indicates that the two forms contain mannose-rich glycans (Figure 1B).

Altogether, the examined Fabry fibroblasts contained a mutation that hampered the protein trafficking of GAA beyond the ER, thus preventing its maturation in the Golgi apparatus and further transport to the lysosomes where it should exert its physiological enzymatic function.

In a previous study, GAA activity in fibroblasts harbouring the N215S mutation could be substantially enhanced by 1-deoxygalactonojirimycin [21].

### Distortion of LRs constitutes a pathomechanism in FD which can be reversed by NB-DNJ treatment

LRs are liquid ordered dynamic, cholesterol- and sphingolipid-enriched assemblies of proteins and lipids, in which cholesterol is arranged between the acyl chains of sphingosine [22]. Imbalance in sphingolipids could lead to distortion of the liquid ordered state and LRs assembly.

The trafficking defect of GAA results in an increased level of cellular sphingolipids in cellular membranes [23]. We, therefore, addressed the question whether this increase is associated with imbalanced lipid microdomains or LRs, which are enriched in sphingolipids and cholesterol.

**Figure 2:**
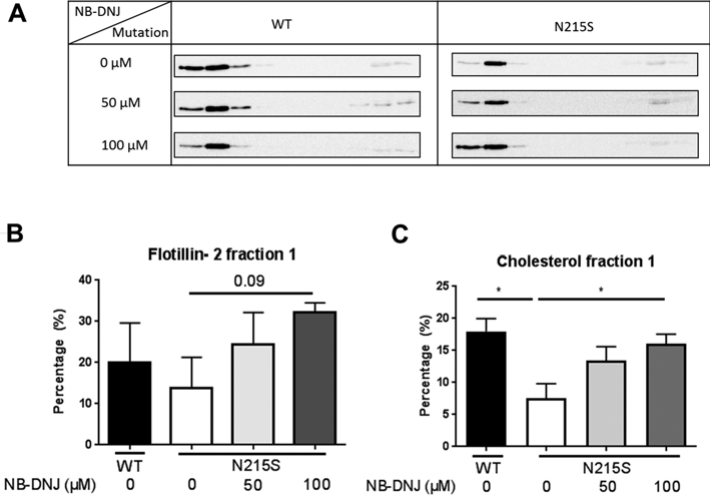
Effect of NB-DNJ on the altered flotillin-2 distribution in LRs in fibroblasts derived from Fabry patients Fibroblasts derived from a Fabry patient (N215S) or age-matched healthy individuals (WT) were treated with NB-DNJ for 3 days and were subsequently lysed with 1% (w/v) Triton X-100 and run on density-based sucrose gradients. Ten fractions were collected and analysed for distribution of flotillin-2 by immunoblotting. (**A**) Immunoblots from a healthy individual (WT) and from a patient (N215S) showing distribution of flotillin-2 in above-mentioned gradient fractions. The mean value + S.E.M. of flotillin-2 or cholesterol distribution in the first floating fraction was calculated (**B**,**C**). The percent of flotillin-2 in the first fraction was 20.1% for the WT and 13.8% for the FD cell line. Post 100 μM NB-DNJ treatment of the FD cell line, the percentage of flotillin-2 altered to 32.2% for fraction 1. (C) Cholesterol analysis of the first fraction performed by HPLC (*P<0.05, S.E.M., *n*=3).

Figure 2A and Supplementary Figure S3 depict Western blot analysis of sucrose gradient fractions using anti-flotillin-2 antibodies. Flotillin-2 protein is associated with LRs and is often used as their protein marker [24]. Flotillin-2 distribution from two age-matched WT cells are displayed in Supplementary Figure S3. Essentially, a biphasic distribution of flotillin-2 among the gradient fractions of the wild-type fibroblasts was revealed (Figure 2A and Supplementary Figure S3), whereby the flotillin-2-rich LRs appear mostly in fraction 2 and to a lesser extent in fraction 1 and 3 (Supplementary Figures S3–S5). Also, the bottom fractions (8–10) revealed flotillin-2 albeit to a substantially lower extent than in the top fractions. Similar to the wild-type pattern, flotillin-2 was also predominantly revealed in fraction 2 of the gradient of FD fibroblasts and minute amounts of flotillin-2 in fractions 1 and 3 (Figure 2A, right panel, top, Supplementary Figures S4 and S5). However, quantification of flotillin-2 in the first fraction is shown in Figure 2B and revealed slightly different percentage of flotillin-2 in fraction 1 between WT and FD fibroblasts. Since WT2 showed level closer to FD fibroblasts (Supplementary Figure S3), this WT was used for all other experiments throughout the manuscript. The amount of flotillin-2 in the first fraction was 20.1% for the WT and 13.8% for the FD cell line (Figure 2B and Supplementary Figure S5).

Similar to flotillin-2, cholesterol levels were also higher in WT than FD fibroblasts, with 18.0 and 7.6% of the total cholesterol present in fraction 1 respectively (Figure 2C, Supplementary Figures S6 and S7). In summary, a good correlation between cholesterol and flotillin-2 distributions of the FD fibroblasts in the first three LR fractions was evident (Supplementary Figures S4–S7), whereby an altered distribution of cholesterol and flotillin-2 comparing WT with FD fibroblasts in fraction 1–3 was detectable (Figure 2B,C, Supplementary Figures 4–7).

Despite this altered distribution within the separated LR fractions 1–3 comparing WT with FD fibroblasts, there was no significant difference in the overall flotillin-2 amount comparing total raft and non-raft fractions of WT and FD fibroblasts (Supplementary Figure S8). To verify that there is no partial diffusion of the specific fractions 1–3, the sucrose concentration was tested (Supplementary Figure S1): after ultracentrifugation, there is still a distinguishable difference in the sucrose concentration between fractions 1 and 2 where lipid and protein distribution was shown to be markedly different between Fabry and wild-type cells. With the sucrose density in fraction 1 being measured at lower than of what has been previously reported for LRs isolated by Triton X-100 (i.e. between 1.060 and 1.090 g/ml or ~15–23% w/v sucrose), this makes comparisons in fractions 1 and 2 between control and treated samples legitimate and reliable [25,26]. Thus, this specific shift of flotillin-2 to a less-dense subfraction in FD fibroblasts (fraction 2, Supplementary Figures S4 and S5) suggests that the character and thereby eventually the function has changed, but not necessarily the amount.

We next examined whether NB-DNJ, as a potential substrate-reducing agent, can restore the flotillin-2 distribution or reverse the LR alterations. NB-DNJ is a glucose analogue inhibitor of ceramide-specific glucosyltransferase that catalyses the first step of glycosphingolipid biosynthesis [27]. Treatment of Fabry cells with 50 or 100 μM NB-DNJ for 72 h resulted in a redistribution of flotillin-2 from the second fraction towards the first fraction in FD fibroblasts thus mimicking the flotillin-2 profile in the untreated wild-type fibroblasts (Figure 2A,B and Supplementary Figures S4 and S5). NB-DNJ treatment also led to a normalization in the distribution of flotillin-2 in both fractions 2 and 3, where upon treatment, the percentage of flotillin-2 decreased in fraction 2 and increased in fraction 3 towards that of the levels present in wild-type fibroblasts. Interestingly, the cholesterol-flotillin-2 correlation was also evident upon NB-DNJ treatment, whereby an increase in cholesterol in fractions 1 and 3 corresponded to a similar increase in flotillin-2 (Figure 2C and Supplementary Figures S6 and S7). Essentially, the distribution pattern of flotillin-2 was restored to normal in the Fabry cells at 100 μM of NB-DNJ. Treatment of WT fibroblasts with NB-DNJ (Figure 2A) led to disruption in LRs, which may be attributed to NB-DNJ inhibiting the biosynthesis of glucosylceramide and subsequently glycosphingolipids. Concomitantly, the cholesterol levels in fraction 1 increased with increasing NB-DNJ concentrations in the Fabry cells (Figure 2C). However, under similar treatment conditions with NB-DNJ in the wild-type cells, a distortion of LRs was observed as determined by the redistribution of flotillin-2 and its substantial reduction in fraction 1 in a fashion similar to the non-treated Fabry cells (Figure 2A, left, lowest panel).

**Figure 3:**
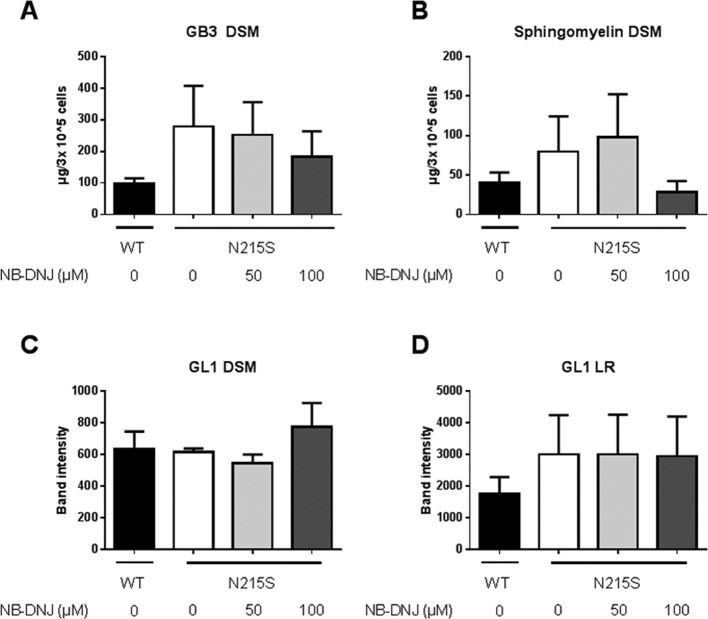
Effect of SRT on membrane glycolipid and SM composition in fibroblasts derived from Fabry patients Glycolipid analysis was performed by orcinol-stained TLC plates of wild-type and a FD fibroblast cell line (**A**, **C** and **D**) and SM analysis by HPTLC (**B**). (A) depicts the concentration of glycolipid Gb3 in DSM fractions. (B) shows the concentration of non-raft (DSM) SM. (C and D) depict the relative amounts of the unidentified glycolipid GL 1 from DSM fractions. All results are presented as a mean + S.E.M. of three to four independent experiments.

Lastly, the concentration and distribution of glycolipids, namely globotriaosylceramide 3 (Gb3) and another unidentified glycolipid (named GL 1) and SM was determined in the non-raft (detergent-soluble membrane, DSM) fractions (Figure 3A–C) and LR fractions (Figure 3D). Distinctly higher concentrations of Gb3 and SM were detected in the non-raft fractions of FD fibroblasts compared with wildtype cells. These results support the hypothesis that FD fibroblasts have a distorted structure of LRs (Figure 2).

Interestingly, upon treatment with 100 μM NB-DNJ, the levels of Gb3 and SM decreased in non-raft fractions, reaching almost WT levels (Figure 3A,B respectively). LR concentrations of GL 1 were slightly higher, but not significantly higher in FD fibroblasts (Figure 3D). Treatment with NB-DNJ led to no significant changes in the concentration of LR GL 1 levels (Figure 3D). Furthermore, no distinct difference of WT and FD fibroblasts in the presence or absence of NB-DNJ for other tested phospholipids (see standard curves, Supplementary Figure S2) were detectable (results not shown). However, since SM and cholesterol are the main components of LRs, the results are therefore compatible with the notion that normalization of the balance of these lipids through NB-DNJ treatment has resulted in a restoration of the LRs.

Taken together, it may be hypothesized that NB-DNJ could play a regulatory role in re-establishing lipid homoeostasis in Fabry cells.

## Concluding remarks

FD is a multisystemic disease [28] and the mutation investigated led to dysfunction of several organs, especially the heart. Pathophysiologically, this can be best explained by compromised function of a basic metabolic pathway.

Our results support the view that disturbances of the glyco- and sphingolipid balance in cell membranes in Fabry fibroblasts are associated with alterations in LRs, which are enriched in these types of membrane lipids. The results shown here are supportive of this concept, since the flotillin-2 distribution appears distorted when compared with the functional wild-type situation. The present study constitutes a pilot experiment, further research will be required based on a larger cohort of patients to prove the hypothesis presented here and to determine the extent of LR alterations modulations and how they correspond to the pathophysiology of the disease.

The observed abnormalities in LRs also affect protein trafficking to outer plasma membrane, as shown previously by Maalouf et al. [7] and further described here for DPPIV with less protein on cell surface in FD fibroblasts (Supplementary Figure S9), supporting the view that LRs are implicated in the trafficking and sorting of a variety of membrane proteins and their distortion, as shown in FD, could play a role in the pathophysiology of other lysosomal storage diseases [29]. The importance of glycosphingolipid homoeostasis in the formation of fully functioning rafts has also been illustrated; whereby the function of LRs in appropriate sorting of its associated proteins to late endosomes, lysosomes and the plasma membrane interconnects lipid- and protein homoeostasis [30]. Thus, failure of glycolipid hydrolysis in the lysosomes leads to an imbalance in membranes, particularly LRs, resulting in protein and lipid maltrafficking which is later elucidated in a broad range of clinical symptoms associated to FD.

It is not completely understood how the failure to hydrolyse a glycolipid in the lysosome elicits distortion of the membrane lipids including LRs. It could be argued that the entrapment of glycolipids along with their metabolites inside the endolysosomes inhibits their recycling back to the membrane, subsequently leading to their reduction at the cell surface during early stages of storage. During the course of disease progression, the storage material later spreads to the rest of the cell compartments to reach the cell membrane. Indeed, several studies have shown that Gb3 accumulates significantly after 2 months in GAA-silenced cells [31] and GAA-knockout mice [32]. In both cases, an imbalance in the lipid composition leads to reduced associations of proteins with LRs.

Presently, enzyme replacement therapy by α-galactosidase A produced in human fibroblasts or Chinese hamster ovary cells is the method of choice for treating FD patients. Recently, 1-deoxygalactonojirimycin (migalastat) was approved by EMA for the treatment of FD in patients harbouring an amenable mutation [33]. Further experimentation regarding lipid composition of biomembranes, protein trafficking and LRs should be performed with this substance in a group of patients harbouring different mutations. This therapeutic option may reduce the levels of SM and Gb3 restoring a balanced LRs composition in terms of cholesterol and flotillin-2 distribution in cellular membranes.

## Compliance with ethics guidelines

The present study was conducted with the approval of the local ethics review board at Hannover Medical School, Germany. Skin biopsies for fibroblast cultures were obtained under local anaesthesia for diagnostic purposes or during routine surgery after informed consent was obtained. The cells were pseudomized.

## Abbreviations

DPPIV: dipeptidyl peptidase IV
DSM: detergent-soluble membrane
EMA: European Medicines Agency
endo H: endoglycosidase H
ER: endoplasmic reticulum
FD: Fabry disease
GAA: α-galactosidase A
Gb3: globotriaosylceramide 3
GL 1: glycolipid 1
HPTLC: high performance TLC
HRP: horseradish peroxidase
LR: lipid raft
NB-DNJ: *N*-butyldeoxynojirimycin
PFA: paraformaldehyde
Rf: retention factor
SM: sphingomyelin
SRT: substrate-reduction therapy
WT: wild type
